# Clinical Implications of Preoperative Thoracic Spine Magnetic Resonance Imaging for Percutaneous Spinal Cord Stimulation Procedures: A Large Retrospective Cohort Study

**DOI:** 10.1111/papr.70211

**Published:** 2026-10-06

**Authors:** Geert Oldenbeuving, Monique C. M. Schel‐Huisman, Jeroen S. Vos, Anastasios Kyriazopoulos

**Affiliations:** ^1^ Department of Anesthesiology and Pain Medicine Erasmus MC University Medical Center Rotterdam the Netherlands; ^2^ Department of Anesthesiology and Pain Medicine St. Maartenskliniek Nijmegen Nijmegen the Netherlands; ^3^ Department of Radiology St. Maartenskliniek Nijmegen Nijmegen the Netherlands

## Abstract

**Introduction:**

Spinal cord stimulation (SCS) is an effective method for pain relief in patients with Persistent Spinal Pain Syndrome (PSPS) type II. International guidelines recommend preoperative imaging to assess spinal anatomy for placement of thoracic SCS leads. This study examines the clinical implications of routine preoperative imaging in patients scheduled for thoracic percutaneous SCS lead placement.

**Methods:**

A single‐center retrospective study of MRIs was performed in patients who were planned for SCS lead placement in a period from January 2020 to March 2023. The study excluded lead revisions and cervical or dorsal root lead placements. We examined the impact of imaging findings on procedural planning and execution, categorizing the clinical impact as minor (additional imaging or consultation) or major (cancellation or technical modification of the procedure). We also evaluated the correlation between abnormalities in the epidural space and periprocedural (but not postprocedural) complications such as dural puncture or improper lead positioning at Th7.

**Results:**

Of the 167 patients who underwent MR imaging, 129 continued with SCS lead placement. The major reason for not proceeding with lead placement was patient and/or physician preference for conservative treatment. MRI revealed abnormalities in 58 patients—22 in the relevant epidural space and 36 incidental findings. In 29 of the patients (17.4% of total) these abnormalities had no clinical consequences. There were minor consequences in 23 patients (13.8% of total) and major consequences in 6 patients (3.6% of total). Major consequences included the decision to perform a primary surgical implantation in one patient, changes in epidural entry level in two patients, surgical decompression in two patients, and cancellation of the procedure in one patient. Of all MRI findings with clinical implications, 14 of 29 (48.2%) (and 3 of 6 for major consequences) were visible on prior imaging. Although not specifically powered for this correlation, statistical analysis using a Fisher exact test found no significant association between epidural abnormalities on MRI and the occurrence of dural puncture, the inability to place the lead at the desired position, or perioperative neurological sequelae.

**Conclusion:**

Routine preoperative MRI before thoracic SCS lead placement often detects abnormalities with major consequences in 3.6% of cases. In half of these cases, the abnormalities could not be identified on previous imaging, all of which were in the thoracic region. There was no association between epidural abnormalities and periprocedural complications in our data.

## 1 Introduction

Spinal cord stimulation (SCS) is a proven effective method for pain reduction in patients suffering from persistent spinal pain syndrome (PSPS) [1, 2]. SCS for leg and back pain consists of placing one or more electrode leads in the epidural space at the mid‐thoracic level. Leads can be placed percutaneously or via a direct surgical approach requiring a laminectomy [3, 4].

International guidelines recommend reviewing preoperative imaging to assess the spinal anatomy at the needle placement for percutaneous placement [5, 6] and assess the anatomy via imaging of the final lead position on a case‐by‐case basis for percutaneous and in all cases for surgical lead placement [6] in order to prevent neurological complications. This is more strongly recommended in patients who are sedated during placement. Neurological injury or dural puncture after percutaneous SCS lead placement is rare [7, 8] with an incidence below 1%.

A recent study showed that for surgical thoracic SCS lead placement, preoperative MRI of the relevant spinal anatomy affects clinical decision making [9]. Twenty‐two percent of patients had MRI findings that had clinical implications (surgical planning) and 3% of patients required additional surgery (surgical decompression). Another recent single‐center study showed that for percutaneous leads preoperative MRIs influenced decision making in 21% of cervical cases and 5% of lumbar/thoracic cases [10].

There is limited clinical evidence for the clinical impact of preoperative imaging on clinical decision making in percutaneous SCS lead placement. Given that an MRI is a patient burden (transportation, time, associated costs) and results in increased demands on the health care system, it is important to weigh the benefits of an MRI against the aforementioned costs.

This study aims to evaluate the clinical impact of preoperative MRI findings for primary percutaneous thoracic SCS lead placement. We evaluate the number of abnormal findings identified by MRI, distinguishing between findings involving the epidural lead tract between the 2nd lumbar vertebra and the 7th thoracic vertebra and incidental findings outside this area. We also look at the association between abnormalities and relevant procedural complications such as inability to place the lead at the correct level, dural puncture, and periprocedural neurological injury. Finally, we will assess whether abnormalities with clinical impact were visible on previously available imaging.

## 2 Materials and Methods

This study was a single‐center, retrospective cohort analysis conducted with clinical data from the Sint Maartenskliniek in Ubbergen, the Netherlands. The study was approved by the local institutional review board. We included all patients who underwent routine MRI of the relevant anatomy (high lumbar to low cervical epidural) as part of the work‐up for primary percutaneous thoracic SCS lead placement. Imaging the low cervical spine in the sagittal plane is standard imaging work‐up in our center and is routinely performed and unrelated to clinical presentation.

Patients undergoing lead revision, dorsal root ganglion (DRG) stimulation or cervical lead placement were excluded from this study. The data collection period was from January 2020 to March 2023.

All cases were discussed preoperatively in a multidisciplinary consultation, involving pain specialists, orthopedic surgeons, radiologists, rehabilitation specialists, neurologists, psychologists, and nurse practitioners. Data for the study were extracted from the reports of these multidisciplinary meetings, MRI imaging and imaging reports, and SCS procedure reports. The MRI scans, sagittal T1, T2 (whole spine) and transverse T1 (Th5‐L3), were performed on a Philips Ingenia 3T and Philips Evolution 3T scanner. MRI reports and images were reviewed by a radiologist, while case discussion and surgery reports were reviewed by a nurse practitioner and an experienced implanter. Patient data were extracted from the electronic hospital information system, stored and managed in Microsoft Excel 2016 (Microsoft Corporation, Redmond, WA, USA). Statistical analysis was performed using RStudio (version 2024.09.0+375, RStudio, PBC, Boston, MA, USA).

We assessed the clinical implication of MRI findings, focusing separately on abnormalities within the procedurally relevant epidural space from the 2nd lumbar vertebra (L2) to the 7th thoracic vertebra (Th7) and incidental findings outside this area. If MRI findings had clinical consequences, they were categorized as minor or major. Due to the nature of our standard preoperative MRI, scanning the upper lumbar and the lower cervical area, it is possible to identify certain lumbar and cervical pathologies.

Minor consequences were defined as those that did not significantly alter the SCS clinical trajectory: requiring additional consultations or referrals to other specialists (such as orthopedic surgeons or neurologists) or requiring further imaging studies, such as a cervical MRI. In these cases, SCS implantation may have been delayed, but the procedure could be continued without changes in the technical approach. When referral resulted in additional surgery (e.g., lumbar, cervical, outside the spine) or the detection of major disease (e.g., cancer), the consequence of the MRI was classified as major.

Major consequences were defined as those that led to substantial changes in the treatment plan: technical changes in the planned procedure, referral for surgical lead placement, additional lumbar surgery or cancellation of the procedure. In our center, we closely collaborate with a neurosurgeon to discuss potential surgical leads. This is done without a referral, but via videoconference with image sharing. If a patient with imaging was discussed (but not referred) with a neurosurgeon for surgical lead placement but was decided on percutaneous lead placement, this was defined as a minor consequence. In addition, we also examined whether clinically significant abnormalities identified on the MRI were present in prior imaging studies that were available at the time of implantation.

Finally, we assessed the relationship between abnormalities detected in the relevant epidural space and the composite risk of dural puncture, the inability to position the lead at the midline of Th7, or periprocedural neurological injury. Periprocedural neurological injury is defined as severe radiating pain following a (multi)radicular pattern that is reported by the patient after the conscious sedation is stopped during the procedure.

To establish a potential association, we used Fisher's exact test with a significance threshold set at a *p*‐value of less than 0.05 under the assumption we would find less than 5 patients in each individual category in the 2 × 2 table.

## 3 Results

During the inclusion period, 167 MRIs were performed in 167 patients and 129 percutaneous SCS procedures were performed. Baseline characteristics for patients screened for SCS are shown in Table 1.

**TABLE 1:** Characteristics of patients screened for SCS (*n* = 167).

Age in years (mean±SD)	60.9 ± 11.5
Sex, *n* (%)	
Female	95 (57.2)
Male	71 (42.8)
BMI (mean±SD)	27.4 ± 4.10
Obesity, *n* (%)	45 (26.6)
No. of spine surgeries (mean±SD)	2.05 ± 1.32
Use of anticoagulants, *n* (%)	35 (20.7)

### 3.1 Proceeding to SCS Trial

Of all patients discussed in the multidisciplinary team and consulted for SCS, 129 patients continued with a SCS trial. Reasons for not continuing with SCS are shown in Table 2.

**TABLE 2:** Characteristics of patients who did not proceed to an SCS trial (*n* = 38).

Medical preference for conservative management (*n*)	11
Patient preference for conservative management (*n*)	18
Surgical decompression (*n*)	2
Deceased (other reason) before implantation (*n*)	1
Other medical disease requiring more urgent treatment (*n*)	5
Medical preference for surgical lead placement (*n*)	1

### 3.2 Incidence of Abnormalities on MRI

A total of 58 (34.7%) of patients had an MRI that showed at least one abnormal finding. The majority of abnormal findings (*n* = 34) were incidental findings. Of the incidental findings the vast majority were of cervical origin (*n* = 23). Other incidental findings were thoracic pathology (*n* = 8) (e.g., arachnoid cyst, disc herniation above Th7) or lower lumbar pathology (*n* = 4) (e.g., central canal stenosis, non‐union of vertebrae after lumbar interbody fusion). Findings in the relevant epidural space (L2–Th7) were seen in 24 (14.3%) of patients. In half of these patients there was a reduction in epidural space at one level and in half of patients there was a reduction in epidural space on multiple levels.

Reasons for reduced epidural space were epidural (post‐surgical) fibrosis, naturally narrow spinal canal because of short pedicles and/or thickened ligamentum flavum, and degenerative changes like facet joint hypertrophy, ligamentum flavum thickening, and/or osteophyte formation.

### 3.3 Clinical Consequences of MRI Abnormalities

In half of the patients with abnormal findings on MRI (*n* = 29), there were no clinical consequences. In 14 of these 29 MRIs the abnormal findings could be identified on prior available imaging.

23 patients had abnormalities on imaging with minor clinical implications: five cases were discussed with (but not referred to) a neurosurgeon for surgical implantation and percutaneous placement was recommended (all successful), two cases were referred to an orthopedic surgeon for lumbar pathology, eight cases were referred to an orthopedic surgeon for cervical pathology, in two cases additional imaging was performed (potential malignant pathology and a spinal cyst), in four cases an additional interdisciplinary meeting was scheduled and in two cases referral to other specialties was done (oncology and internal medicine). 11 out of 23 imaging abnormalities with minor consequences could be identified on older available imaging. In the case of potential malignancy, no malignancy was detected. In the case of the spinal cyst, no treatment was deemed necessary.

In six patients (3.6%), the findings had significant clinical consequences, four of which were related to findings in the relevant epidural space, as shown in Table 3. One patient was referred for surgical implantation after consultation with and referral to a neurosurgeon, two patients had a change in epidural entry level because of previous high lumbar surgery, and one case was canceled because of multilevel thoracic canal stenosis for which referral to a neurologist and conservative management was preferred over SCS (resulting in cancellation of the procedure). In two patients, incidental findings in the lumbar region resulted in surgical decompression at the lumbar level. Half of the imaging abnormalities with major implications could be identified on prior available imaging. All the imaging abnormalities with major consequences which could be seen on prior imaging were of lumbar origin. There were no abnormalities in the cervical region with major clinical consequences.

**TABLE 3:** Findings in the relevant epidural space with major consequences on MRI (*n* = 4).

MRI finding	Type of major consequence	Visible on prior imaging
Central stenosis L1‐L2 with reduced epidural space and prior surgery with facet joint degeneration at L2	Epidural entry one level higher	Yes
Central stenosis L1‐L2 with reduced epidural space due to flavum hypertrophy	Epidural entry one level higher	Yes
Central stenosis with reduced epidural space at Th10‐L2, concomitant progressive cervical canal stenosis	Cancellation of percutaneous procedure, referral to neurologist.	No
Minimal epidural space Th10‐Th11 due to vascular structure	Referral for surgical lead, cancellation of percutaneous procedure	No

### 3.4 Periprocedural Complications

There were no major neurological sequelae during implantation, but there were two dural punctures and eight cases where a lead could not be placed at the desired location (midline Th7) (Table 4). All patients had one or multiple leads implanted during the procedure. In patients in whom a spinal tap occurred, there were no abnormalities of the relevant epidural space on MR imaging. In 3 out of 8 patients in whom the lead could not be positioned at the desired location, there were abnormalities of the epidural space on MR imaging.

**TABLE 4:** Overview of complications and possible relationship with MRI (*n* = 8).

Complication	Level of complication	MRI abnormalities	Relation between MRI and complication
Unable to position lead at Th7 level	Th9	Minimal epidural stenosis at Th9‐Th11	Yes
Unable to position lead in midline	Th10	None	No
Unable to position lead the Th7 level	Th7	None	No
Unable to position lead at Th7 level	Th9	None	No
Unable to position lead at Th7	Th8	None	No
Spinal tap	L1	None	No
Spinal tap	L1	None	No
Unable to position lead at Th7	Th9	None	No
Unable to position lead in midline	Th9/Th10	Multilevel epidural stenosis, most prominent at Th9‐Th10	Yes
Unable to position lead in midline	Th9/Th10	Multilevel epidural stenosis, most prominent at Th9‐Th10	Yes

Statistical analysis using a Fisher exact test showed no significant difference between imaging abnormalities in the relevant epidural space and the composite outcome of periprocedural neurological sequelae, dural puncture, and inability to place the lead at midline Th7 (*p* = 0.127).

## 4 Discussion

The objective of this study was to assess the clinical impact of routine preoperative MRI on the placement of thoracic percutaneous SCS leads. Our findings suggest that preoperative MRI identifies abnormalities with clinical implications.

Abnormalities were detected on MRI in 58 (34.7%) patients. The majority (*n* = 34) of abnormal findings were incidental findings outside the relevant epidural space (L2–Th7). The preoperative MRI had major consequences in 6 patients (3.6% of total). In half of these patients, the MRI abnormality was visible on prior imaging, all in the lumbar region.

Our study emphasizes the need for critical evaluation of available imaging of the lumbar region before proceeding with SCS. If these imaging abnormalities were identified and discussed with a neurosurgeon or orthopedic surgeon before proceeding with SCS and the concomitant MRI, time and resources may be saved.

Even if these cases were identified before preoperative MRI, it would have yielded an important change in the clinical trajectory in 1.8% of patients for percutaneous SCS lead placement. All these abnormalities were based on abnormalities in the thoracic region.

Since there are not many indications for thoracic spine imaging, and many for lumbar or cervical imaging, this finding may be expected. Interestingly, in the only case with major consequences due to any cervical pathology, the cancellation of the procedure was based on thoracic canal stenosis. All the incidental findings in the cervical regions did not have major clinical consequences, but it should be noted that the cervical spine was only scanned in the sagittal plane.

Although not specifically designed and powered to assess the correlation, there was no statistical correlation between MRI‐detected abnormalities in the relevant epidural space and the composite outcome of dural puncture, periprocedural neurological sequelae, and inability to place the lead at the Th7 midline. The majority (7 out of 10) of patients with dural puncture or inability to place the lead at the Th7 midline had no abnormalities on imaging. It is important to stress that we did not screen for postoperative neurological sequelae due to limited resources.

We cannot assess the effect of available imaging on the risk of such complications; identifying abnormalities may help prevent complications by increasing awareness of where periprocedural problems may arise.

### 4.1 Limitations

This study has important limitations. First, our study is a single‐center retrospective analysis, the findings may be biased and not generalizable to other institutions or patient populations. Furthermore, the applicability of our results to other procedures may be limited by the exclusion of patients undergoing revisions or other types of lead placements (e.g., DRG, cervical lead). This study was not designed or powered to assess the predictive value of imaging on complications. Our study only looked at periprocedural neurological complications and did not look at postoperative neurological injury because of limited resources.

## 5 Conclusion

In our dataset, performing an MRI had major clinical consequences in 3.6% of patients. In half of these cases, the abnormalities could not be identified on previous imaging, all of which were in the thoracic region.

We recommend careful consideration of available imaging before proceeding to preoperative imaging specific for percutaneous SCS lead placement.

Finally, with the emphasis that our study was not designed for the detection of neurologic complications, there was no statistical correlation between MRI abnormalities and periprocedural complications.

## Author Contributions

All authors have made substantial contributions to the conception, design, analysis, or interpretation of the work. They have been actively involved in drafting or critically revising the manuscript, are fully acquainted with the study's content, and have approved the final version for submission. Each author willingly accepts responsibility for the integrity and accuracy of the article.

## Funding

The authors have nothing to report.

## Ethics Statement

The study was approved by the local institutional review board.

## Consent

In this retrospective study, informed consent was not required as it did not involve direct patient contact.

## Conflicts of Interest

The authors declare no conflicts of interest.

## Data Availability

The data used in this study are available upon request from the corresponding author.
